# Brain Swelling versus Infarct Size: A Problematizing Review

**DOI:** 10.3390/brainsci14030229

**Published:** 2024-02-28

**Authors:** J. Marc Simard, Bradley Wilhelmy, Natalya Tsymbalyuk, Bosung Shim, Jesse A. Stokum, Madison Evans, Anandita Gaur, Cigdem Tosun, Kaspar Keledjian, Prajwal Ciryam, Riccardo Serra, Volodymyr Gerzanich

**Affiliations:** 1 Department of Neurosurgery, University of Maryland School of Medicine, Baltimore, MD 21201, USA; bradley.wilhelmy@som.umaryland.edu (B.W.); ntsymbalyuk@som.umaryland.edu (N.T.); bosung.shim@som.umaryland.edu (B.S.); jstokum@som.umaryland.edu (J.A.S.); madison.evans@som.umaryland.edu (M.E.); anangaur@terpmail.umd.edu (A.G.); ctosun@som.umaryland.edu (C.T.); kkeledjian@som.umaryland.edu (K.K.); rserra@som.umaryland.edu (R.S.); vgerzanich@som.umaryland.edu (V.G.); 2 Department of Pathology, University of Maryland School of Medicine, Baltimore, MD 21201, USA; 3 Department of Physiology, University of Maryland School of Medicine, Baltimore, MD 21201, USA; 4 Department of Neurology, University of Maryland School of Medicine, Baltimore, MD 21201, USA; pciryam@som.umaryland.edu

**Keywords:** brain swelling, brain edema, stroke, cerebral ischemia, middle cerebral artery occlusion, SUR1-TRPM4, review

## Abstract

In human stroke, brain swelling is an important predictor of neurological outcome and mortality, yet treatments to reduce or prevent brain swelling are extremely limited, due in part to an inadequate understanding of mechanisms. In preclinical studies on cerebroprotection in animal models of stroke, historically, the focus has been on reducing infarct size, and in most studies, a reduction in infarct size has been associated with a corresponding reduction in brain swelling. Unfortunately, such findings on brain swelling have little translational value for treating brain swelling in patients with stroke. This is because, in humans, brain swelling usually becomes evident, either symptomatically or radiologically, days after the infarct size has stabilized, requiring that the prevention or treatment of brain swelling target mechanism(s) that are independent of a reduction in infarct size. In this problematizing review, we highlight the often-neglected concept that brain edema and brain swelling are not simply secondary, correlative phenomena of stroke but distinct pathological entities with unique molecular and cellular mechanisms that are worthy of direct targeting. We outline the advances in approaches for the study of brain swelling that are independent of a reduction in infarct size. Although straightforward, the approaches reviewed in this study have important translational relevance for identifying novel treatment targets for post-ischemic brain swelling.

## 1. Introduction

Acute ischemic stroke is a leading cause of death and disability worldwide [1]. Morbidity and mortality in ischemic stroke are determined in part by secondary injury, the most important in the acute phase being brain swelling. In a stroke of moderate severity, brain swelling is an independent predictor of poor outcome [2,3]. Among patients with large hemispheric infarction, those who develop severe or “malignant” cerebral edema with massive brain swelling consistently exhibit high inpatient mortality, high utilization of health resources, and high costs [4]. Malignant cerebral edema is associated with poor clinical outcomes (mRS ≥ 3) [5], it places patients at high risk for neurological deterioration and is largely responsible for the high mortality rate of 50–80% in large hemispheric infarction [6].

Approved treatments for brain edema/swelling are limited. There is a long history of the use of hyperosmolar therapies, including mannitol and hypertonic saline, in ischemic stroke [7,8]. Although both agents can reduce intracranial pressure and may increase cerebral blood flow in non-ischemic tissues acutely [9,10], neither agent was shown to improve patient outcomes in a randomized clinical trial [11]. Moreover, hyperosmolar therapies usually are administered only after brain swelling has already compromised neurological function, oftentimes irreversibly. The only proven treatment for severe brain swelling is decompressive craniectomy, which is associated with a considerable reduction in death or severe disability [12]. However, decompressive craniectomy, which involves the surgical removal of a large part of the cranium, is also associated with non-trivial morbidity [13].

In decades of preclinical work on ischemic stroke models, the primary goal in characterizing potential cerebroprotectants has typically been to reduce infarct size. The closely linked outcomes of brain edema and brain swelling are usually treated as secondary correlative phenomena. During its two decades of important work, the Stroke Therapy Academic Industry Roundtable (STAIR) has provided numerous recommendations that highlight critical issues in the experimental modeling of ischemic stroke, including study design, therapeutic drug dose, the choice of animal model, outcome measures, anesthesia protocol, physiological monitoring, and many others, as recently updated [14]. However, little emphasis has been placed on the study of post-ischemic cerebral edema or brain swelling as a distinctly targetable, druggable disease process.

In animal models and humans, it is generally considered that brain swelling is proportional to infarct volume [15]. Innumerable preclinical studies have reaffirmed this dictum, showing that treatments that reduce infarct size almost invariably are accompanied by a reduction in brain edema/swelling. Unfortunately, such findings on brain swelling have little translational value for treating brain swelling in patients with stroke. This is because, in humans, brain swelling typically peaks 3–5 days after stroke onset [16], well after the infarct size has stabilized and thus the opportunity to reduce swelling by reducing infarct size has passed. This requires that the treatment of brain swelling target mechanism(s) that are independent of a reduction in infarct size. The clinical management of stroke requires molecularly informed treatments to reduce brain swelling, especially with large infarcts [17], without the requirement to reduce infarct size.

Here, we present a problematizing review [18,19] that emphasizes an unconventional way of thinking about brain edema/swelling in preclinical work—not simply as a secondary correlative phenomenon but as a distinct pathological entity worthy of targeting separately from the infarct. Based on a review of the literature on middle cerebral artery occlusion (MCAo) models, we first identify several drug and gene candidates that have been reported to reduce edema disproportionately compared to a reduction in infarct size. We then demonstrate the utility of specific rigorous approaches that may be utilized to confirm the involvement of a given pathway in post-ischemic brain swelling that is independent of a reduction in infarct size.

## 2. Materials and Methods

We searched PubMed (16 January 2024) using the terms “edema [title] AND (middle cerebral artery occlusion OR MCAO) AND mouse”, which yielded 65 citations. Abstracts or full-length papers were screened for reports on the effects of treatment (pharmacological or genetic) on brain edema or swelling. Additional citations were identified from the references cited in these papers and from our publications. Reports were excluded if (1) an effect on edema/swelling was not reported; (2) both infarct and edema were significantly reduced by treatment; (3) edema was affected, but an effect on infarct size was not reported. This process yielded reports with statistically significant effects on edema/swelling (usually a reduction) with no statistically significant effect on infarct size.

## 3. Results

We first sought to identify the potential targets and treatments for edema/swelling that might act via a mechanism independent of a reduction in infarct size. We searched the literature for examples of treatments, either pharmacological or genetic, that would affect edema/swelling in the absence of a significant effect on infarct size. Our search identified several treatments that had a disproportionate effect on edema/swelling compared to the effect on infarct size (Table 1) and thus might be considered potential candidates for reducing edema independent of infarct size. These included glibenclamide [20,21]; dimethyl fumarate (Tecfidera^®^) [22] and its metabolite monomethyl fumarate [23]; soluble programmed death ligand-1 [24]; and canagliflozin [25]. All of these drugs reduced edema/swelling without significantly reducing infarct size when administered post-ischemia in MCAo models. A similar effect of reducing edema/swelling without a significant reduction in infarct size was reported with the global knockout of several genes, including *Trpm4*/TRPM4 (transient receptor potential melastatin 4) [21], *Grin2C*/GluN2C (glutamate receptor, ionotropic) [26], and *Verge*/Verge (vascular early response gene) [27], as well as with the astrocyte-specific knockout of *Abcc8*/SUR1 (sulfonylurea receptor 1) and *Slc8a1*/NCX1 (sodium–calcium exchanger 1) [21]. Conversely, worsened edema with no significant effect on infarct size was reported in fingolimod [28], the transgenic expression of *Elavl1*/HuR (human antigen R) in astrocytes [29], and the global knockout of *P2rx7*/P2X7 (purinoceptor 7) [30] or *Slc1a2*/GLT-1 (solute carrier family 1 member 2) [31]. 

The above list of drugs and genes was compiled based on reports of no statistically significant effect of treatment on infarct size. Overall, this is a useful metric for identifying the potential mechanisms of swelling that may be independent of infarct size. Possible limitations include (1) instances in which a moderate reduction in infarct size may have been observed, but the study lacked the power to detect statistical significance; and (2) instances in which a moderate ischemic insult of relatively short duration was used, which led to a large variance in infarct sizes. Below, we present the approaches that may be utilized to overcome such limitations and strengthen any claim that a drug or gene may be directly involved in regulating post-ischemic brain edema/swelling independent of infarct size.

Two approaches are discussed: (1) plotting the data for brain swelling vs. infarct size over the entire range of infarct sizes for both treated and control subjects, and determining whether the data from the control group and the treatment group are best fit with a single function or with two functions; (2) comparing swelling between two groups with equivalent (large, non-reducible) infarcts following a severe ischemic insult. In both approaches, if data on hemispheric swelling for treated subjects are different from controls, one can reasonably conclude that the treatment is targeting a mechanism of swelling that is independent of infarct size. Below, we present examples from the literature published by our group and others, supplemented by our unpublished observations.

### 3.1. Swelling as a Function of Infarct Size—Does Treatment Change the Relationship?

**Figure 1:**
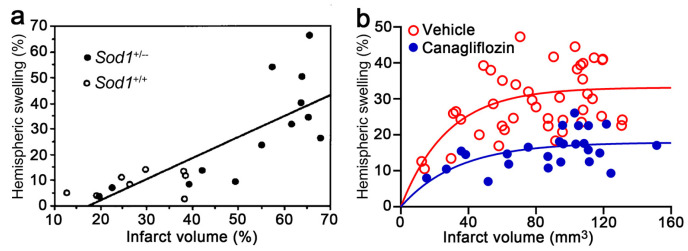
Brain swelling as a function of infarct size—does the relationship change with genotype or treatment? (**a**) Plot of hemispheric swelling as a function of infarct size over the range of infarct sizes for two genotypes. Data for individual wild-type (*Sod1*^+/+^) (*empty circles*, *n* = 8) and heterozygous (*Sod1*^+/−^) (*filled circles*, *n* = 13) mice are shown. Visual inspection reveals no apparent change in relationship due to genotype. Linear regression analysis of the combined datasets showed a positive correlation between the percentage of infarct volume and the percentage of hemisphere enlargement (*r* = 0.80); male mice with MCAo/R (1/24 h); from Kondo et al. [32] with permission (copyright 1997, Society for Neuroscience). (**b**) Plot of hemispheric swelling as a function of infarct size over the range of infarct sizes for both treated and control subjects. Data for individual vehicle-treated (*empty circles*, *n* = 43) and canagliflozin-treated (*filled circles*, *n* = 25) mice are shown. Visual inspection reveals an apparent change in relationship due to treatment. The relationship between hemispheric swelling and infarct volume was analyzed using the equation Y = Y_M_ [1 − exp(−k × X)], where X is infarct volume (mm^3^), Y is hemispheric swelling (%), Y_M_ is the maximum hemispheric swelling (%), and k is the rate of change (1/mm^3^). Non-linear least-square fit of each dataset yielded values of k = 0.030/mm^3^ vs. 0.036/mm^3^, and Y_M_ = 17.9% vs. 33.2% (*p* < 0.0001) for canagliflozin vs. vehicle, respectively; male mice with MCAo/R (2/24 h); data derived in part from Shim et al. [25] with permission (copyright 2023, the authors) and from unpublished data.

It is well known that the mouse MCAo/reperfusion (MCAo/R) model of stroke is associated with significant variability in infarct size [32], and it has long been recognized that there is a good correlation between the volume of brain swelling and the volume of infarction (*r* = 0.73, *p* < 0.05) [15]. Thus, plotting brain swelling as a function of infarct size for a given treatment vs. control can be informative. A clear example of this comes from a study by Kondo et al. [33], in which the authors studied a mouse model of MCAo lasting 1 h followed by 24 h reperfusion (MCAo/R, 1/24 h). These authors examined the effect of the global knockout of CuZn–superoxide dismutase (SOD) 1 (*Sod1*) in heterozygous mice. Data on hemispheric enlargement vs. infarct size for *Sod1*^+/−^ and *Sod1*^+/+^ mice are shown in Figure 1a. A visual inspection of the scatter plot indicates that there is no obvious change in the relationship of swelling to infarct size due to genotype. The combined data from the two groups are well fit with a single function, consistent with a decrease in the level of CuZn–SOD in *Sod1*^+/−^ mice exacerbating both infarction and swelling in a proportional manner, with no independent effect on brain swelling. Since the treatment did *not* change the relationship between swelling and infarct size, one can reasonably conclude that the treatment is not targeting a mechanism of brain swelling that is independent of infarct size.

A similar analytical approach of plotting brain swelling vs. infarct size was used to analyze data from a mouse model (MCAo/R, 2/24 h) in which mice were administered vehicle vs. canagliflozin, an inhibitor of sodium/glucose cotransporter 2 (SGLT2), at reperfusion (data from Shim et al. [25] and unpublished data). Data on hemispheric swelling vs. infarct size are shown in Figure 1b. In this example, data from all subjects are plotted, including those with small infarcts that would normally have been excluded due to inadequate reduction in relative cerebral blood flow or some other reason. Unlike the experiment in Figure 1a, the data presented in Figure 1b are not linear, possibly because a more severe ischemia model was used. Also, data from the two groups in Figure 1b do not superimpose well, i.e., they appear to be distinct. The latter observation was confirmed by a fit of the two datasets to an equation with plateau values that were statistically different (Figure 1b, legend). In both groups, swelling increased with infarct size, but in the group treated with the drug, swelling plateaued at a lower level than in controls, consistent with an effect of drug treatment on brain swelling that is independent of infarct size. Since the treatment *did* change the relationship between swelling and infarct size, one can reasonably conclude that the treatment is targeting a mechanism of swelling that is independent of infarct size.

### 3.2. Swelling with Equivalent (Large) Infarct Sizes in Both Groups

**Figure 2:**
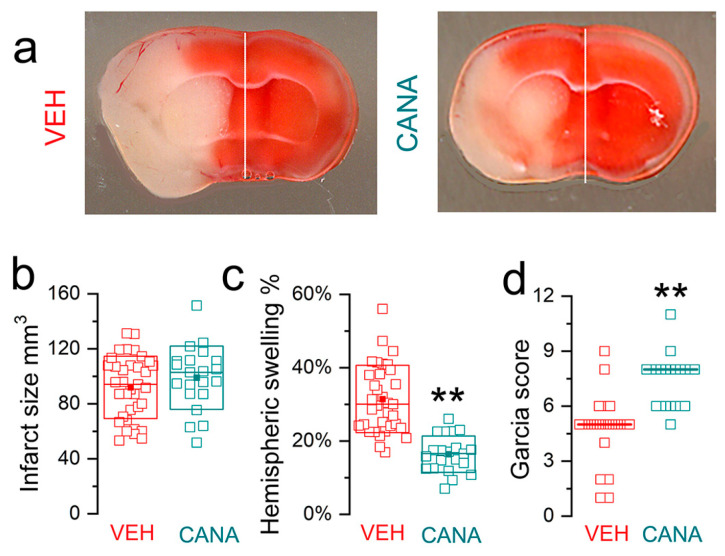
Brain swelling in subjects with large (non-reducible) infarcts, highlighting the significant effect of treatment: (**a**) Images at 24 h of TTC (2,3,5-triphenyltetrazolium chloride)-stained coronal sections from mice administered vehicle (VEH) vs. canagliflozin (CANA) at reperfusion. (**b**–**d**) Plots of infarct size (**b**), hemispheric swelling (**c**), and Garcia scores (**d**) for subjects with large infarcts, after excluding subjects with infarcts <40 mm^3^. Data for individual vehicle-treated (*n* = 35) and canagliflozin-treated (*n* = 21) mice are shown. Analysis of infarct volumes showed no difference between groups. Analysis of hemispheric swelling showed a significant difference between VEH and CANA, as did the analysis of Garcia scores; ** *p* < 0.01; male mice with MCAo/R (2/24 h); from Shim et al. [25] with permission (copyright 2023, the authors).

Figure 1b further illustrates that data on brain swelling from subjects with large infarcts may be sufficient to distinguish between treatment and control. Based on this observation, data from the same experimental series as above were analyzed after excluding subjects from both the control and treatment groups that had infarcts < 40 mm^3^ (determined empirically from Figure 1b), leaving only subjects with large infarcts [25]. This selection process yielded two groups with infarct sizes that were not different (Figure 2a,b), thus establishing a meaningful baseline for the comparison of brain swelling between the groups. In accordance with the findings from Figure 1b, hemispheric swelling was decreased by drug treatment (Figure 2c), consistent with an effect of drug treatment on brain swelling that is independent of infarct size. Unsurprisingly, the reduction in swelling was accompanied by an improvement in neurological function, as measured by Garcia scores (Figure 2d), despite equivalent infarct sizes.

An important advantage of the second approach (large infarcts only) compared to the first approach (an entire range of infarct sizes) is that, with the former, a smaller number of subjects may be adequate to identify an effect on swelling. It is relatively straightforward to implement a severe ischemia model and then exclude subjects with small infarcts, aiming for the two groups with large infarcts that are not different in size. Excluding subjects with small infarcts is experimentally similar to excluding subjects with inadequate reductions in relative cerebral blood flow during occlusion, a selection criterion that has been universally adopted in preclinical studies. The MCAo/R (2/24 h) mouse model is well suited to this type of experiment since mortality was minimal at 24 h (although it increased sharply thereafter). Rigor and reproducibility were maintained by an objective, automated analysis of 2,3,5-triphenyltetrazolium chloride (TTC)-stained coronal brain sections and by the blinded identification of subjects that met the prespecified threshold of infarct size (40–50 mm^3^) for inclusion. TTC-stained coronal brain sections were processed using the NIH ImageJ software (version 1.52a) with a semi-automated plug-in, which features automatic thresholding that yields unbiased measurements of TTC-negative tissue areas and hemisphere areas used for calculating volumes [34]. Infarct volumes were calculated, and corrections for edema were performed as described [21].

The methodology focused on selecting large infarcts was implemented in a recent study on SUR1-TRPM4 and its role in post-ischemic brain swelling [21]. Multiple genotypes and drugs were studied to establish the multi-step signaling pathway culminating in aquaporin-4 membrane trafficking that underlies brain swelling. For all groups, the same stroke model was used (MCAo/R, 2/24 h), and infarct and hemispheric volumes were measured using the objective, automated algorithm mentioned above. In addition, the same prespecified exclusion volume was used (40 mm^3^), and exclusions for small infarcts were performed in a masked fashion.

**Figure 3:**
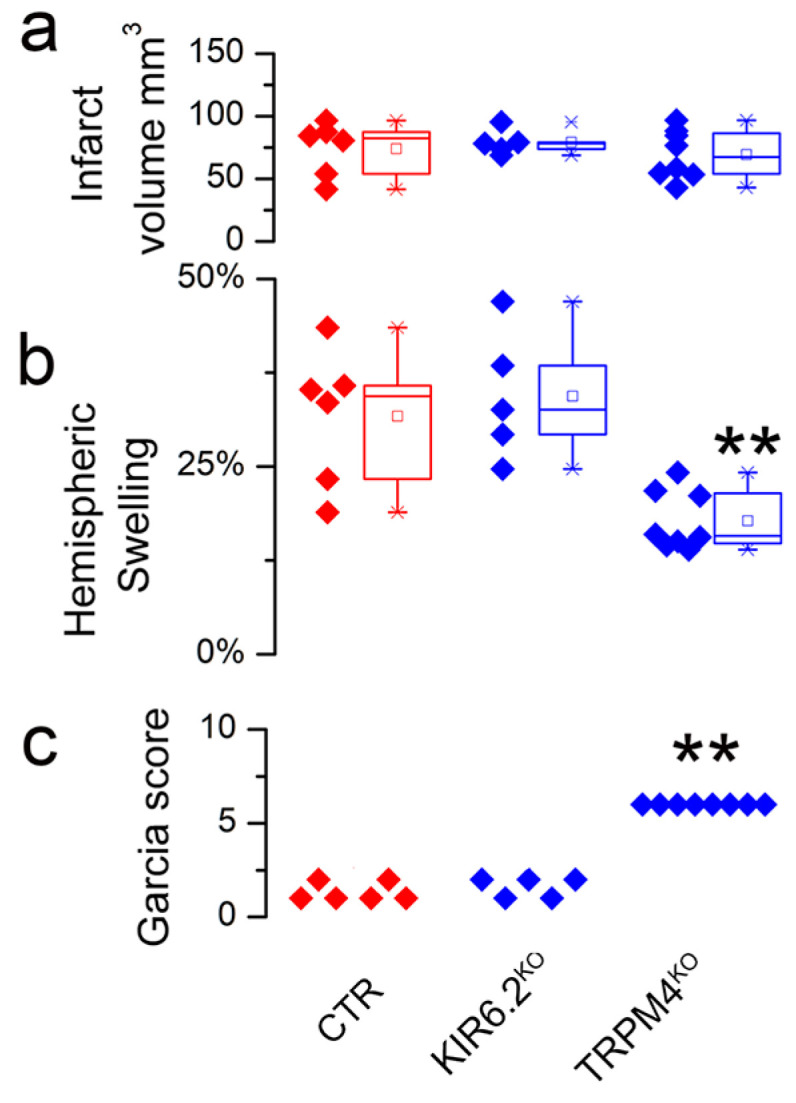
Brain swelling in subjects with large (non-reducible) infarcts—no effect vs. significant effect of genotype: (**a**–**c**) Plots of infarct size (**a**), hemispheric swelling (**b**), and Garcia scores (**c**) for subjects with large infarcts, after excluding subjects with infarcts <40 mm^3^. Data for individual wild-type (CTR) (*n* = 6), KIR6.2^−/−^ (*n* = 5), and TRPM4^−/−^ (*n* = 8) mice are shown. Analysis of infarct volumes showed no difference between groups. Analysis of hemispheric swelling showed no difference between CTR and KIR6.2^−/−^ but a significant difference between CTR and TRPM4^−/−^, as did the analysis of Garcia scores; ** *p* < 0.01; male mice with MCAo/R (2/24 h); from Stokum et al. [21] with permission (copyright 2023, the authors).

In one part of the study, two strains of global knockout mice, KIR6.2^−/−^ and TRPM4^−/−^, were compared. Data on hemispheric swelling vs. infarct size for wild-type (CTR), KIR6.2^−/−^, and TRPM4^−/−^ mice are shown in Figure 3. Infarct volumes were not different between the groups (Figure 3a). Hemispheric swelling was not different in the KIR6.2^−/−^ mice but was decreased in the TRPM4^−/−^ mice (Figure 3b), consistent with an effect of TRPM4 but not KIR6.2 on brain swelling that is independent of infarct size. The reduction in swelling in TRPM4^−/−^ mice was accompanied by better neurological scores (Figure 3c). These findings illustrate that focusing on data from subjects with large infarcts can be a powerful tool in identifying the genes involved, or not involved, in brain swelling.

**Figure 4:**
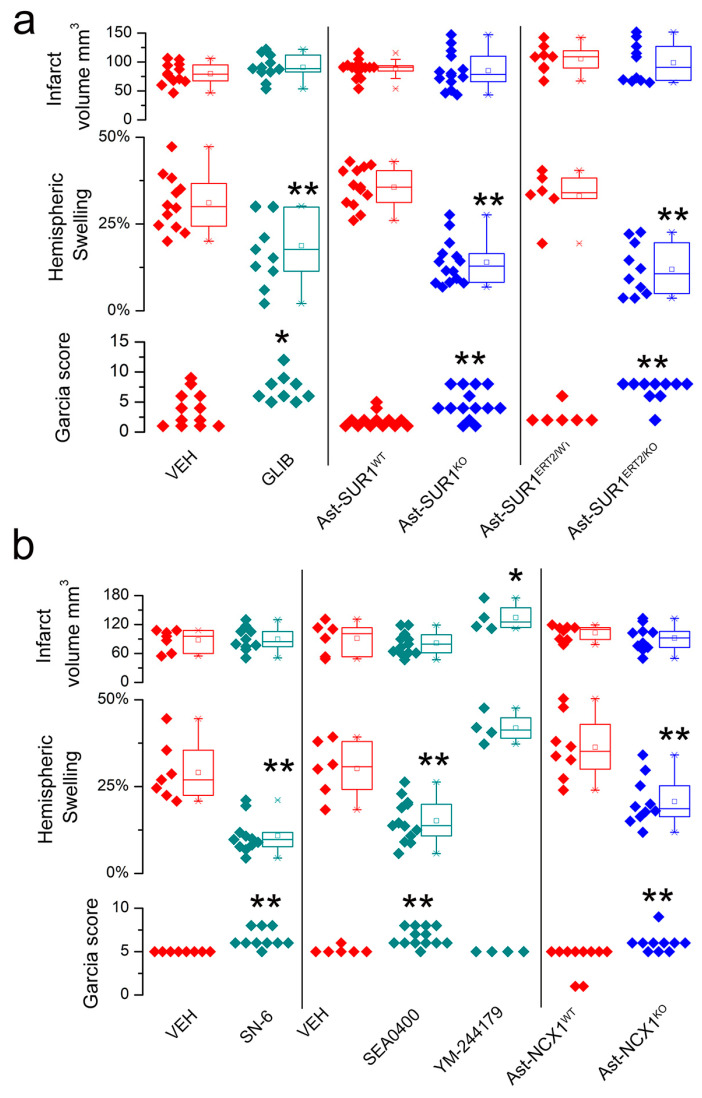
Brain swelling in subjects with large (non-reducible) infarcts—no effect vs. significant effect of treatment or genotype: (**a**,**b**) Plots of infarct size (upper), hemispheric swelling (middle), and Garcia scores (lower) for subjects with large infarcts, after excluding subjects with infarcts < 40 mm^3^. Data for individual vehicle-treated (VEH) vs. glibenclamide-treated (GLIB); wild-type (Ast-SUR1^WT^, Ast-SUR1^ERT2/WT^) vs. astrocyte-specific deletion of SUR1 (Ast-SUR1^KO^, Ast-SUR1^ERT2/KO^); vehicle-treated (VEH) vs. SN-6- or SEA0400- or YM-244179-treated; and wild-type (Ast-NCX1^WT^) vs. astrocyte-specific deletion of NCX1 (Ast-NCX1^KO^) mice are shown; 4–14 mice/group. With the exception of YM-244179, analysis of infarct volumes showed no difference between groups, whereas analysis of hemispheric swelling showed a significant difference between controls and treatments, as did the analysis of Garcia scores; * *p* < 0.05; ** *p* < 0.01; male mice with MCAo/R (2/24 h); from Stokum et al. [21] with permission (copyright 2023, the authors).

Further evidence of the utility of this approach comes from another part of the same report [21] in which the astrocyte-specific gene deletion of *Abcc8*/SUR1 or *Slc8a1*/NCX1, as well as several drugs targeting these gene products, were studied. Here, group sizes were 4–14 mice/group. Infarct volumes were not different between the groups (Figure 4a,b, upper rows). Again, the pathways involved or not involved in brain swelling were clearly identified. YM-244179, which targets NCX3, did not reduce swelling, whereas in the other six of the seven paired comparisons, hemispheric swelling was reduced by either cell-specific gene deletion or drug treatment (Figure 4a,b, middle rows), consistent with an effect of these treatments on brain swelling independent of infarct size. The reductions in swelling were accompanied by better neurological function, as reflected in better Garcia scores (Figure 4a,b, lower rows).

A refinement to this approach in which there is no effect of treatment on infarct volume was presented by Kim et al. [24]. These authors studied the effect of soluble programmed death ligand-1 (sPD-L1) in a mouse MCAo (0.75/48 h) model using high-resolution magnetic resonance imaging. Infarct volumes were not statistically different between the control and treated groups. However, the mice treated with sPD-L1 had significantly lower volumes of edema (Table 1). The authors of this study utilized an MCAo/R model with relatively brief ischemia (45 min) that gave rise to considerable variance in infarct size. To account for the marked variance in infarct volumes, the authors normalized the data to obtain the “volume of edema per volume of infarct”. This calculation yielded a variable with greatly reduced variance and allowed for a definitive statement about a statistically significant reduction in edema with treatment.

The study by Kim et al. [24] not only illustrates the utility of normalization but also underscores the importance of studying an MCAo model with severe ischemia. As noted previously, it is well known that the mouse MCAo/R model of stroke is associated with significant variability in infarct size [32], especially with the moderate ischemic insults that are frequently utilized. However, with severe (prolonged) ischemia, infarcts are more likely to be uniformly large and not be reducible by treatment—the ideal situation for studying mechanisms of edema independent of infarct size.

## 4. Discussion

In human stroke, brain swelling that occurs early on is an independent predictor of clinical outcome measured at 90 days and beyond [2,3]. Understanding the mechanisms, treatment, and prevention of brain edema/swelling early after stroke is of great clinical importance and requires a careful study of appropriate animal models.

In preclinical studies on rodent models of stroke, innumerable pharmacological or genetic treatments have been identified that reduce both infarct size and edema/swelling [35]. However, unless the effects on edema vs. infarct are markedly disproportionate, the treatments in these reports cannot be unequivocally said to have targeted a mechanism directly linked to edema, since edema decreases with a reduction in infarct size. Similarly, numerous treatments have been identified that reduce edema/swelling, but many of these reports lack critical information about infarct size as a baseline comparator. The most promising reports on the effects of a given treatment on edema/swelling are those in which a reduction in edema/swelling is observed independent of infarct size, either over the entire range of infarct sizes or with large, irreducible infarcts.

Similar considerations apply to neurological function since neurological function is directly linked to infarct size [36]. Reports of improved neurological function accompanying a reduction in both infarct size and edema/swelling or accompanying a reduction in edema/swelling but with no data on infarct size are ambiguous as to the cause. The most promising reports linking neurological function to edema/swelling are those in which treatment effects are independent of infarct size, preferably with large, irreducible infarcts, as reviewed with treatments in Table 1. In these cases, it is reasonable to conclude that improved neurological function was linked to edema/swelling, highlighting the critical role that edema and swelling play in determining neurological function.

For some of the treatments identified in Table 1, additional studies have reported that the same treatment reduced both infarct size and edema/swelling. Such was reported for glibenclamide [21,37,38], dimethyl fumarate [39,40], and canagliflozin [25]. In our experience, this apparent discrepancy is not problematic and, in some cases, is deliberate, resulting from the careful selection of models. In MCAo/R models with moderate ischemic insults (e.g., 1 h ischemia in the mouse model [21,25]; 105 min in the rat model [38]), a given treatment can reduce both infarct size and edema/swelling, whereas with a severe ischemia model (e.g., 2 h ischemia in the mouse model [21,25]; 4.5 h ischemia in the rat model [20]), especially when subjects with small infarcts are deliberately excluded, the same treatment can reduce edema/swelling but not infarct size. The latter situation provides the ideal context to determine whether a given treatment affects edema/swelling independent of infarct size, which is a prerequisite for studying the molecular and cellular mechanisms involved.

Ultimately, the validation of the concepts and methodologies reviewed here to study brain swelling independent of infarct size will come from clinical studies on stroke patients. In the case of SUR1-TRPM4, mid-phase clinical data on patients with large hemispheric infarctions suggest that early treatment with glibenclamide can reduce midline shift, a standard measure of brain swelling [41]. Ongoing research on drugs for use in humans will determine whether glibenclamide [42] or other drugs can be advanced for the treatment of patients with stroke whose neurological well-being is adversely affected by brain swelling.

## Figures and Tables

**Table 1:** Potential candidates for reducing edema/swelling independent of infarct size, based on the reported disproportionate reduction in edema/swelling vs. infarct size ^1^.

	Reference	Species	MCAo/R	Method	Change in Infarct Size	Change in Edema/Swelling
Glibenclamide	Simard et al. [20]	rat	4.5/24 h ^2^	TTC	−3.2%	−45%
Glibenclamide	Stokum et al. [21]	mouse	2/24 h ^3^	TTC	+12%	−40%
Dimethyl fumarate	Kunze et al. [22]	mouse	1/24 h ^4^	MRI	−6.5%	−70%
Monomethyl fumarate	Clausen et al. [23]	mouse	MCA coagulation	toluidine blue	−1%	−31%
Soluble programmed death ligand-1	Kim et al. [24]	mouse	0.75/72 h ^2^	MRI	−21%	−73%
Canagliflozin	Shim et al. [25]	mouse	2/24 h ^3^	TTC	+9.6%	−45%
TRPM4^−/−^	Stokum et al. [21]	mouse	2/24 h ^3^	TTC	−5%	−56%
GluN2C^−/−^	Holmes et al. [26]	mouse	1.5/24 h ^2^	TTC	−21%	−45%
Verge^−/−^	Liu et al. [27]	mouse	1.5/24 h ^4^	TTC	+10%	−33%
Ast-SUR1^−/−^	Stokum et al. [21]	mouse	2/24 h ^3^	TTC	−4%	−60%
Ast-NCX1^−/−^	Stokum et al. [21]	mouse	2/24 h ^3^	TTC	−10%	−45%

^1^ In all cases, effects on infarct size were reported to be not statistically significant, whereas effects on edema/swelling were statistically significant. ^2^ The patency of the internal and common carotid arteries was restored at reperfusion. ^3^ The common carotid artery was ligated at reperfusion. ^4^ The status of the carotid arteries at reperfusion was not reported.

## Data Availability

No new data were created in this review. All relevant information will be fully shared upon reasonable request to the corresponding author.
